# Living Bacteria‐Based Immuno‐Photodynamic Therapy: Metabolic Labeling of *Clostridium butyricum* for Eradicating Malignant Melanoma

**DOI:** 10.1002/advs.202105807

**Published:** 2022-03-11

**Authors:** Leilei Shi, Xiaoxiao Liu, Yuzhen Li, Sha Li, Wenbo Wu, Xihui Gao, Bin Liu

**Affiliations:** ^1^ Department of Chemical and Biomolecular Engineering National University of Singapore 4 Engineering Drive 4 Singapore 117585 Singapore; ^2^ The Eighth Affiliated Hospital Sun Yat‐Sen University 3025 Shennan Middle Road Shenzhen 518033 China; ^3^ Key Laboratory of Medical Molecular Virology (MOE/NHC/CAMS) School of Basic Medical Sciences Fudan University 131 Dong An Road Shanghai 200032 China

**Keywords:** *Clostridium butyricum*, immunotherapy, melanoma, metabolic labeling, photodynamic therapy

## Abstract

Due to the complexity, aggressiveness, and heterogeneity of malignant melanoma, it is difficult to eradicate the whole tumor through conventional treatment. Herein, a strategy of metabolic engineering labeled anaerobic oncolytic bacteria (*Clostridium butyricum*) is demonstrated to achieve the ablation of melanoma. In this system, the metabolic substrate of *C. butyricum* d‐alanine (d‐Ala) is first conjugated with a photosensitizer (TPApy) showing aggregation‐induced emission (AIE). The yielded metabolic substrate of d‐Ala‐TPAPy can be metabolically incorporated into bacterial peptidoglycan to form engineered *C. Butyricum*. Once the engineered *C. butyricum* is injected into melanoma, the bacteria can only proliferate in an anaerobic zone, stimulate the tumor immune microenvironment, and ablate the tumor hypoxia region. Following that, the relatively rich oxygen content in the peripheral area can induce the death of *C. butyricum*. The photosensitizer (PS) on the bacteria can subsequently exert a photodynamic effect in the oxygen‐rich region and further remove the melanoma residue under light irradiation. Prominent in vivo melanoma ablation results revealed that the engineering oncolytic bacteria can provide a promising regime for solid tumor eradication.

## Graphical abstract

A bacteria metabolic substrate is designed and metabolically incorporated into peptidoglycan to form engineered Clostridium butyricum. After being injected into melanoma, the bacteria can only proliferate in an anaerobic zone, the photosensitizer on the bacteria can subsequently exert a photodynamic effect in the oxygen‐rich region and further remove the melanoma residue under light irradiation.

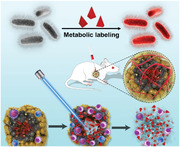

## 1 Introduction

Malignant melanoma is one of the most aggressive malignant tumors, with unpredictable evolution.^[^ ^1^ ^]^ Despite numerous therapeutic options, such as surgery, chemotherapy, v‐raf murine sarcoma viral oncogene homolog B1 (BRAF) inhibitors, and immunotherapy, eradicating the whole tumor through conventional treatment remain unsolved.^[^ ^2^ ^]^ The main reason is that malignant melanoma exhibited low cell adhesion, hematogenous metastasis could easily occur during surgery.^[^ ^3^ ^]^ In addition, the complicated microenvironment, especially the hypoxia microenvironment, would induce tumor relapse and drug resistance.^[^ ^4^ ^]^ As a result, developing a new strategy to eradicate melanoma that avoids surgery and simultaneously overcome drug resistance in the hypoxia region is highly desirable in the field of malignant melanoma treatment.

It is well known that therapeutic agents are difficult to penetrate the hypoxia area of melanoma owing to the lack of blood vessels in this region.^[^ ^5^ ^]^ Additionally, hypoxia melanoma cells are in a dormancy state and exhibit stem cell‐like properties, so that conventional agents could not perform their efficiency on hypoxia melanoma cells.^[^ ^6^ ^]^ However, the local anaerobic microenvironment in the tumor hypoxia region provides the necessary conditions for anaerobic bacteria.^[^ ^7^ ^]^ Anaerobes can compete with tumor cells for nutrients in the hypoxia microenvironment, then restrict tumor growth and cause tumor cells lysis.^[^ ^8^ ^]^ At the same time, the inherent pro‐inflammatory ability of bacteria could also stimulate the adjacent immune system.^[^ ^9^ ^]^ These characteristics of anaerobes provide a promising strategy to utilize living oncolytic bacteria for malignant melanoma treatment.

Generally, the ideal oncolytic bacteria should perform the following characteristics, high targeting capacity, low side effects, strong compatibility, high sensitivity, and good stability.^[^ ^10^ ^]^ Recent studies showed that *Clostridium* exhibited the most significant oncolytic effect in contrast to other types of bacteria.^[^ ^11^ ^]^ *Clostridium* belongs to obligate anaerobes that only propagate in anoxic or necrotic areas of solid tumors so that its side effect on normal tissues is very low.^[^ ^12^ ^]^ However, the limitation of *Clostridium* for melanoma treatment is that the high oxygen concentration in the peripheral rich vascular area of melanoma could induce the death of *Clostridium* to result in an incomplete oncolytic effect at the tumor edge. Although *Clostridium* could not completely eradicate malignant melanoma, the remaining region has a relatively high O_2_ concentration, which provides a good condition for photodynamic therapy (PDT). PDT could perform better ablation in contrast to traditional chemotherapy, as chemotherapy usually could only exert the efficiency to high proliferation rate tumor cells.^[^ ^13^ ^]^ Therefore, by combining the advantages of living bacteria treatment and photodynamic therapy, there stands a chance to achieve complete ablation of malignant melanoma.

**Scheme 1:**
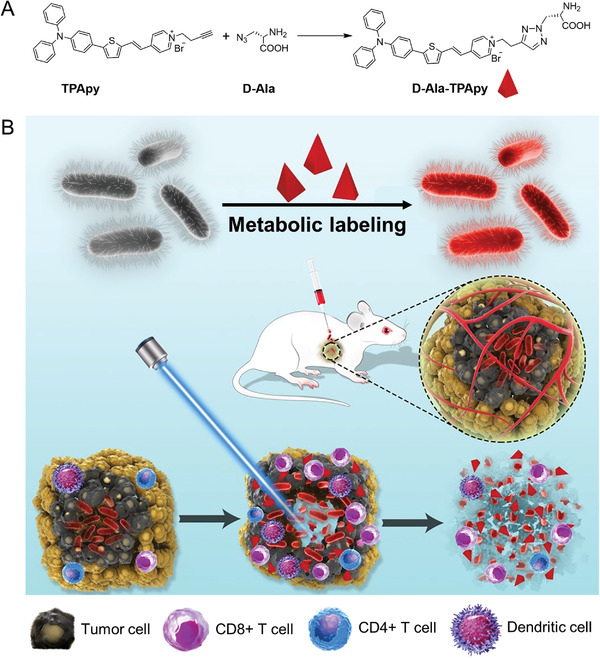
Schematic illustration for the construction of dye‐labeled oncolytic bacteria and melanoma eradicating mechanism by engineering bacteria under light irradiation. a) Synthesis and preparation of metabolic labeling substrate d‐Ala‐TPApy. b) Schematic illustration of d‐Ala‐TPApy‐labeled *Clostridium butyricum* for malignant melanoma ablation under light irradiation.

To demonstrate the idea of metabolic engineering labeled oncolytic bacteria strategy, a bacteria metabolic substrate d‐alanine (d‐Ala) was conjugated with aggregation‐induced emission (AIE) photosensitizer (PS) (TPApy) via click chemistry to prepare d‐Ala‐TPApy (**Scheme** **1**a).^[^ ^14^ ^]^ We chose AIE PSs because they can be designed to show both bright fluorescence and strong photosensitization.^[^ ^15^ ^]^ When d‐Ala‐TPApy was incubated with oncolytic bacteria (*Clostridium butyricum*), TPApy could be precisely and quantitatively incorporated in *C. butyricum* to produce engineered oncolytic bacteria. Theoretically, after intratumoral injection, the engineered *C. butyricum* can ablate the hypoxic region. With the continuous ablation of the melanoma hypoxia area, the oxygen partial pressure gradually increases, and the *C. butyricum* would gradually die to stimulate tumor immune microenvironment and increase immune cell infiltration (Scheme 1b). Since the tumor peripheral is rich in O_2_, it is suitable for PS to exert a photodynamic effect. Therefore, under light irradiation, the peripheral area of malignant melanoma could be further removed by singlet molecular oxygen (^1^O_2_) and immune cells. In this regard, the whole tumor could be completely ablated by the engineered *C. butyricum* (Scheme 1b).

## 2 Results and Discussion

The preparation of d‐Ala‐TPApy is shown in Scheme S1 (Supporting Information). Briefly, Compound 2 was produced via Knoevenagel condensation reaction between compounds 1 and 5‐(4‐(diphenylamino)phenyl)thiophene‐2‐carbaldehyde, which was further conjugated with d‐Ala through copper(I)‐catalyzed azide‐alkyne cycloaddition reaction to yield d‐Ala‐TPApy. The structures of the key intermediates and final product (d‐Ala‐TPApy) were confirmed by mass spectroscopy and NMR (Figures S1–S5, Supporting Information) and the purity was determined by reverse‐phase high‐performance liquid chromatography (HPLC; Figure S6, Supporting Information).

**Figure 1:**
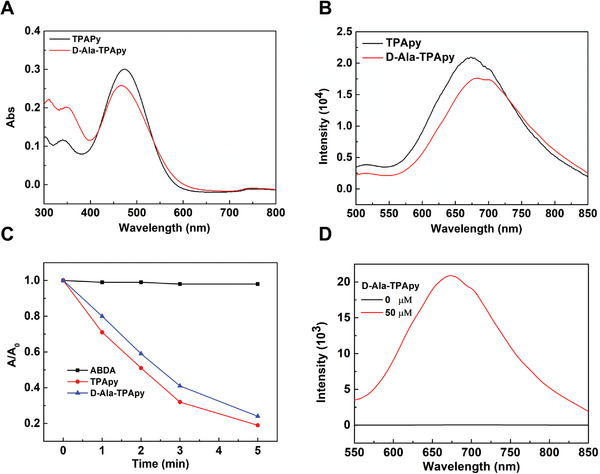
a) UV–vis absorption and b) fluorescence emission spectra of TPApy and d‐Ala‐TPApy. c) Decomposition rates of ABDA induced by ^1^O2 generation from TPApy and d‐Ala‐TPApy ([TPApy] = [d‐Ala‐TPApy] = 10 × 10^−6^ m) in water. d) Fluorescence intensity of *Clostridium butyricum* before and after being incubated with the metabolic substrate (d‐Ala‐TPApy with the concentration of 0 and 50 × 10^−6^ m, respectively).

TPApy possesses a donor–*π*–acceptor (D–*π*–A) structure,^[^ ^16^ ^]^ which displays a maximum absorption peak at 480 nm and an emission peak located at 680 nm in DMSO/water (v/v = 10/90) mixed solution (**Figure** **1**a,b). After being conjugated with d‐Ala, the fluorescence of d‐Ala‐TPApy shows a slight bathochromic shift with a maximum emission peak at 690 nm in DMSO/water (v/v = 1/99) solution (Figure 1b). The ^1^O_2_ generation capacity of both TPApy and d‐Ala‐TPApy was determined by 9,10‐anthracenediyl‐bis(methylene)dimalonic acid (ABDA). As shown in Figure 1c, the degradation rate of ABDA was ≈80% by TPApy and d‐Ala‐TPApy under the same light irradiation (100 mW cm^−2^, 5 min), indicating the highly effective ^1^O_2_ production capability of TPApy. In addition, to study the photosensitive abilities of TPApy core, its overall ROS production efficiency was investigated by dichlorofluorescein (DCFH), whose green fluorescence can be sensitively triggered by any type of ROS. The result showed that along with the continuous irradiation of light, the fluorescence intensity of DCFH enhanced rapidly in the presence of TPApy (Figure S7, Supporting Information). In contrast, no significant fluorescence signal was detected for the solution with TPApy under dark (Figure S7, Supporting Information). The ROS type was further measured by utilizing other indicators. First, the •OH generation was investigated by hydroxyphenyl fluorescein (HPF) as an indicator, which could emit green fluorescence upon reaction with •OH. As shown in Figure S7 (Supporting Information), almost no fluorescence signal could be detected during 5 min light irradiation, demonstrating the low •OH production abilities of TPApy. However, when TPApy was incubated with •O^2−^ indicator HKSOX‐1, the fluorescence intensity was gradually increased with the extension of illumination time (Figure S7, Supporting Information). The results demonstrated that TPApy could product both •O^2−^ and ^1^O_2_ under light irradiation, as a result, we think TPApy could perform both Type‐I and Type‐II reactions under light illumination.

We next studied whether d‐Ala‐TPApy could be utilized to label *C. butyricum* by the metabolic pathway. After incubating *C. butyricum* with d‐Ala‐TPApy for 30 min, *C. butyricum* could be effectively labeled with clear fluorescence enhancement (Figure 1d). The confocal study also showed that the bacteria were easily labeled by TPApy after *C. butyricum* was incubated with d‐Ala‐TPAPy (50 × 10^−6^ m) for 30 min (Figure S8, Supporting Information). Then, mass spectrum (MS) analysis was conducted to confirm the metabolic labeling process. The peptidoglycan of d‐Ala‐TPApy treated and natural *C. butyricum* was isolated by the standard protocol and then determined by matrix‐assisted laser desorption ionization–time of flight mass spectrometry (MALDI–TOF MS). The results showed that unmodified *C. butyricum* has an *m*/*z* (mass‐to‐charge ratio) peak at 942.6, which belongs to the normal peptidoglycan fragment. However, different from unmodified *C. butyricum*, after metabolic labeling, the molecular weight of peptidoglycan was increased from 942 to 1532.8, indicating that TPApy was incorporated into peptidoglycan via metabolic reactions (Figure S9, Supporting Information).

**Figure 2:**
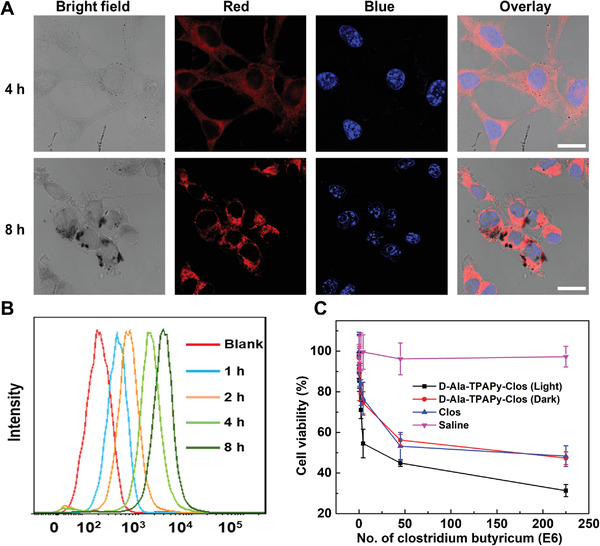
a) Confocal fluorescence images of live B16F10 after treatment with d‐Ala‐TPApy‐labeled *Clostrsidum butyricum* ([d‐Ala‐TPApy] = 10 × 10^−6^ m, 500 µL) for 4 and 8 h. The blue fluorescence indicates cell nuclei stained by Hoechst (Ex: 405 nm, Em: 420–450 nm). The red fluorescence is from TPApy (Ex: 488 nm; Em: 680–750 nm). The scale bar in all the images is 10 µm. b) Cell uptake study after B16F10 cell lines were incubated with d‐Ala‐TPApy‐labeled *Clostrsidum butyricum* at different time intervals. c) Cytotoxicity study of B16F10 cell lines after being treated by saline, unmodified *Clostridium butyricum*, d‐Ala‐TPAPy‐Clos under dark and light illumination, respectively.

As living bio‐therapeutic systems, the cellular uptake capacity of TPApy‐labeled *C. butyricum* (d‐Ala‐TPAPy‐Clos) is critical for disease treatment. Therefore, the tumor cell uptake efficiency of d‐Ala‐TPAPy‐Clos was evaluated by a confocal study. After B16F10 cell lines were incubated with d‐Ala‐TPAPy‐Clos (20 × 10^−6^ m) and d‐Ala‐TPAPy alone (20 × 10^−6^ m), in contrast to free d‐Ala‐TPAPy, the intracellular fluorescence intensity of d‐Ala‐TPAPy‐Clos group was much stronger, demonstrating that TPApy‐labeled *C. butyricum* could be easily uptaken by melanoma cells (**Figure** **2**a,b; Figure S10, Supporting Information). In vitro cytotoxicity study was studied after B16F10 cells were incubated with different drug groups for 72 h. As shown in Figure 2c, d‐Ala‐TPAPy‐Clos could induce cell death under dark, demonstrating efficient tumor cytotoxicity effect of *C. butyricum*. Further, when d‐Ala‐TPAPy‐Clos was incubated with B16F10 cells under light illumination, it could exert an excellent antitumor effect with an IC_50_ value of 1.27 ± 0.038 × 10^−6^ m based on the concentration of TPApy, which is much more significant than d‐Ala‐TPApy alone under light irradiation (Figure S11, Supporting Information). Next, (2’,7’‐dichlorodihydrofluorescein diacetate (DCFDA)), a ROS detection probe was utilized for B16F10 cells staining to detect ROS concentration inside the B16F10 cells. Quantitative flow cytometry results showed that melanoma cells being incubated with TPApy‐labeled bacteria could emit the strongest green fluorescence under light (100 mW cm^−2^, 5 min) (Figure S12, Supporting Information). However, green fluorescence was much weaker for the groups under dark conditions, indicating that TPApy performed the highest intracellular ^1^O_2_ generation efficiency under light irradiation.

**Figure 3:**
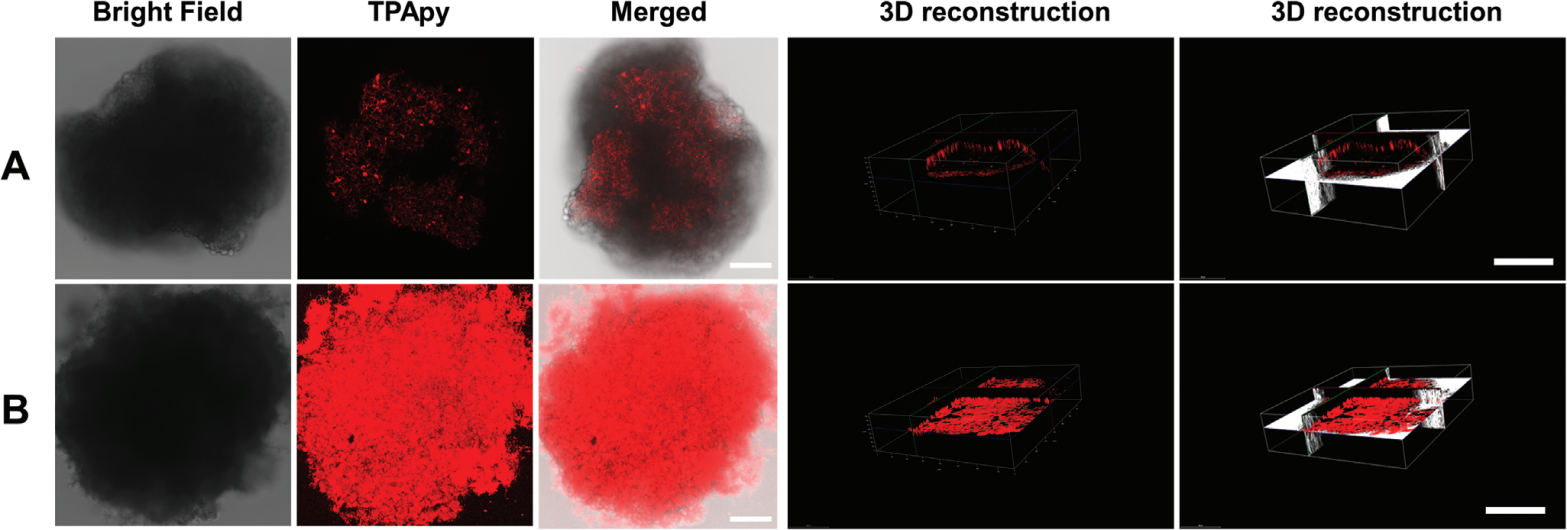
Confocal image study of 3D tumorspheres for tumor penetration evaluation after tumorspheres being incubated with a) d‐Ala‐TPApy (20 × 10^−6^ m) and b) d‐Ala‐TPAPy‐Clos (20 × 10^−6^ m). The scale bar in all the images is 50 µm.

Then, we studied the tumor penetration and tumor eradication capacity of the d‐Ala‐TPApy and d‐Ala‐TPAPy‐Clos in a 3D spheroid model (3DSM) that was originated from B16F10 cancer cells. The 3DSM was constructed by cultivating the cells (5000 per well) in a 24‐well plate, before seeding the cells, dried agarose gel was added at the bottom of each well.^[^ ^17^ ^]^ The cross‐sectional images from both horizontal and vertical directions are shown in **Figure** **3** and Figure S13 (Supporting Information), respectively. d‐Ala‐TPAPy‐Clos could enter into a deeper position of tumor spheroids than free d‐Ala‐TPApy. This result reveals that the anaerobic property of *C. butyricum* makes the d‐Ala‐TPApy labeled Clos penetrate the core region of the solid tumor.

To further evaluate the tumor inhibition efficiency of d‐Ala‐TPAPy‐Clos, 3DSMs were treated with d‐Ala‐TPApy and d‐Ala‐TPAPy‐Clos, respectively, and the size variations were measured over 4 days. As shown in Figure S14 (Supporting Information), free d‐Ala‐TPApy under dark conditions did not perform any significant tumor inhibition effect. Both *C. butyricum* and d‐Ala‐TPAPy‐Clos (under dark) exhibited obvious tumor inhibition with the inhibition rate of 72.3 ± 2% and 75.6 ± 2%, respectively, notably much more effective in contrast to that of d‐Ala‐TPApy under light irradiation (100 mW cm^−2^, 5 min). In contrast, when 3DSMs were incubated with d‐Ala‐TPAPy‐Clos (under light), the tumorspheres were almost totally inhibited with a tumor inhibition rate of 91.7 ± 3%. This result denotes that *C. butyricum* could enter the core region of tumorspheres and destroy the hypoxia region. The photosensitizer (TPApy) could further induce cellular apoptosis of the peripheral area under light irradiation.

**Figure 4:**
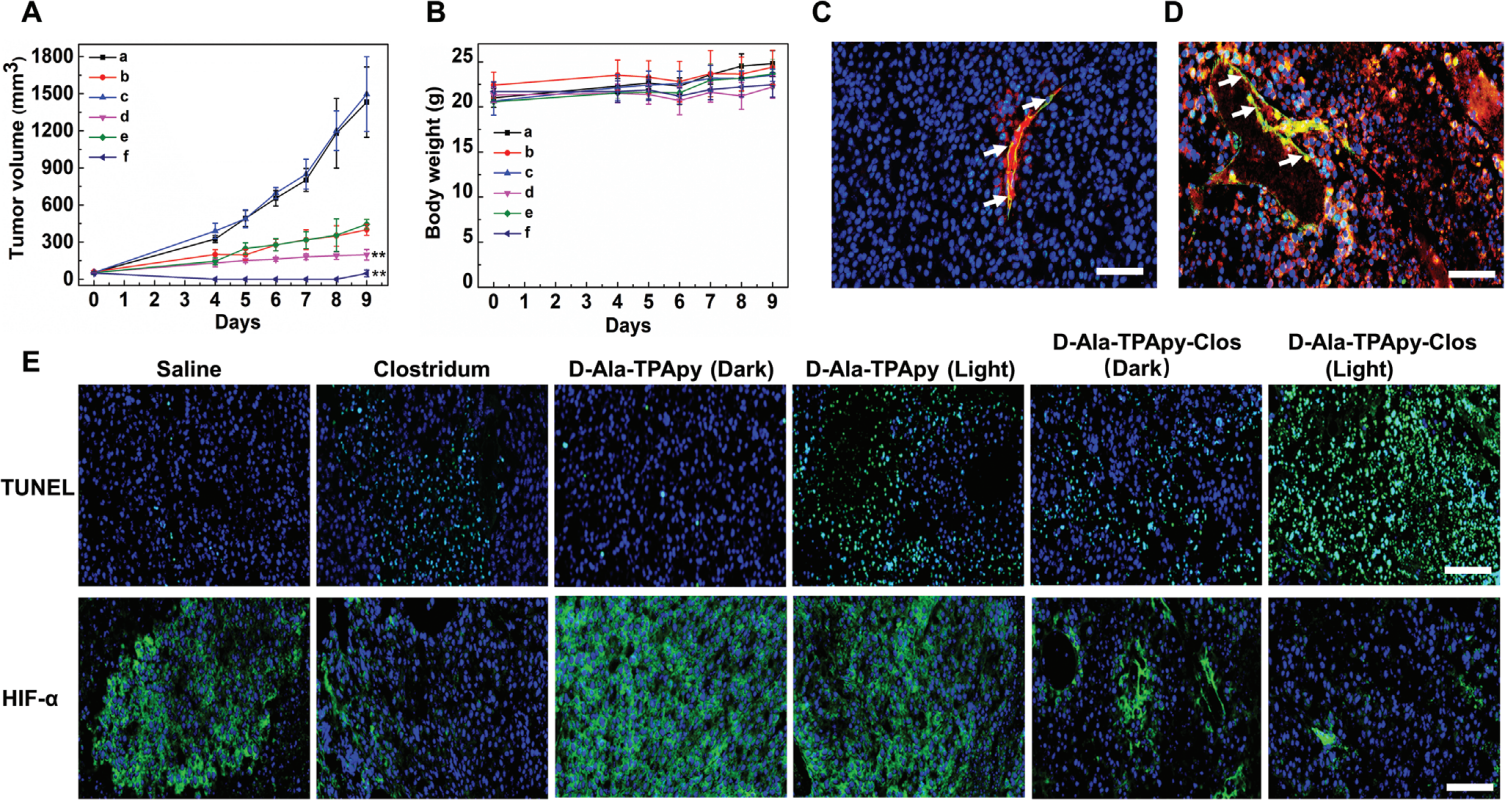
In vivo antitumor study in B16F10 bearing C57BL/6 mice. a) Tumor volume change after administration of saline, b,c) d‐Ala‐TPApy with or without light (100 mW cm^‐2^, 5 min; 5 mg kg^‐1^, 40 µL), d) unmodified *Clostridium*, e,f) d‐Ala‐TPApy‐Clos (5 mg kg^‐1^ based on d‐Ala‐TPApy, 40 µL) with or without light (100 mW cm^‐2^, 5 min). The data presented here was mean ±SD, *n* = 7. The statistical significance level is ^**^ *p* < 0.01. b) Bodyweight change analysis of tumor‐bearing mice. c,d) In vivo tumor penetration study after tumor‐bearing mice being injected d‐Ala‐TPApy (c) and d‐Ala‐TPApy‐Clos (d), respectively. The white arrow indicated blood vessels. Green: the blood vessel; Blue: DAPI stained nuclei; Red: the fluorescence of TPApy. e) Histological immunofluorescence in tumor sites labeled by TUNEL and HIF‐*α*. Green: apoptosis cells or HIF‐*α*. Blue: DAPI‐stained cell nuclei. Scale bar is 50 µm.

In vivo antitumor efficiency and tumor penetration of different formulations were further investigated through a mouse malignant melanoma model. The mice carried with melanoma were administrated with different formulations through one‐time intratumoral injection. The volume of melanoma in saline and TPApy groups under dark raised from ≈110 to 1500 mm^3^ and the bodyweight of the two groups was without any significant change, indicating that TPApy performs negligible toxicity under dark (**Figure** **4**a,b). As shown by hematoxylin/eosin (H&E) analysis, the melanoma tissues in both saline and TPApy groups under dark did not induce significant damage to melanoma cells (Figure S15, Supporting Information). When melanoma mice were treated with *C. butyricum* (4.0 × 10^8^, 50 µL) and d‐Ala‐TPApy‐Clos (4.0 × 10^8^, 50 µL) without light, tumor growth was suppressed because *C. butyricum* could exert an antitumor effect (Figure 4a,b; Figure S16, Supporting Information). For the TPApy (30 µg mL^−1^, 50 µL) group under light irradiation (100 mW cm^−2^, 5 min), tumor growth was greatly inhibited, demonstrating that TPApy exhibited effective phototoxicity under light irradiation. The most prominent eradication effect could be found after being treated by d‐Ala‐TPApy‐Clos (4.0 × 10^8^, 50 µL) under light irradiation, where in vivo melanoma volume was almost completely inhibited and body weight did not show any obvious change (Figure 4a,b; Figure S16, Supporting Information). In the meantime, a large number of dead tumor cells with disappeared nuclei could be observed by H&E staining, and d‐Ala‐TPApy‐Clos group (light) exhibited the supreme apoptosis and antiproliferation rate, as confirmed by the TUNEL study (Figure 4e; Figures S15–S17, Supporting Information). To further identify the capacity of *C. butyricum* to destroy the hypoxia region, immunofluorescence analysis was used to determine the expression level of hypoxia‐inducible factor‐1*α* (HIF‐1*α*) by fluorescent‐dye‐labeled antibody staining. Compared to the saline and d‐Ala‐TPApy groups, *C. butyricum* groups showed weak green fluorescence (Figure 4e), indicating the HIF‐1*α* expression was significantly reduced. It was because that hypoxia region of melanoma could be dissolved by our *C. butyricum*. Eventually, no damage could be found in normal organs after mice were administrated with d‐Ala‐TPApy, *C. butyricum*, and d‐Ala‐TPApy‐Clos, respectively (Figure S18, Supporting Information). Overall, after d‐Ala‐TPApy‐Clos treatment under light irradiation, a prominent in vivo melanoma eradication effect could be achieved with good biocompatibility.

**Figure 5:**
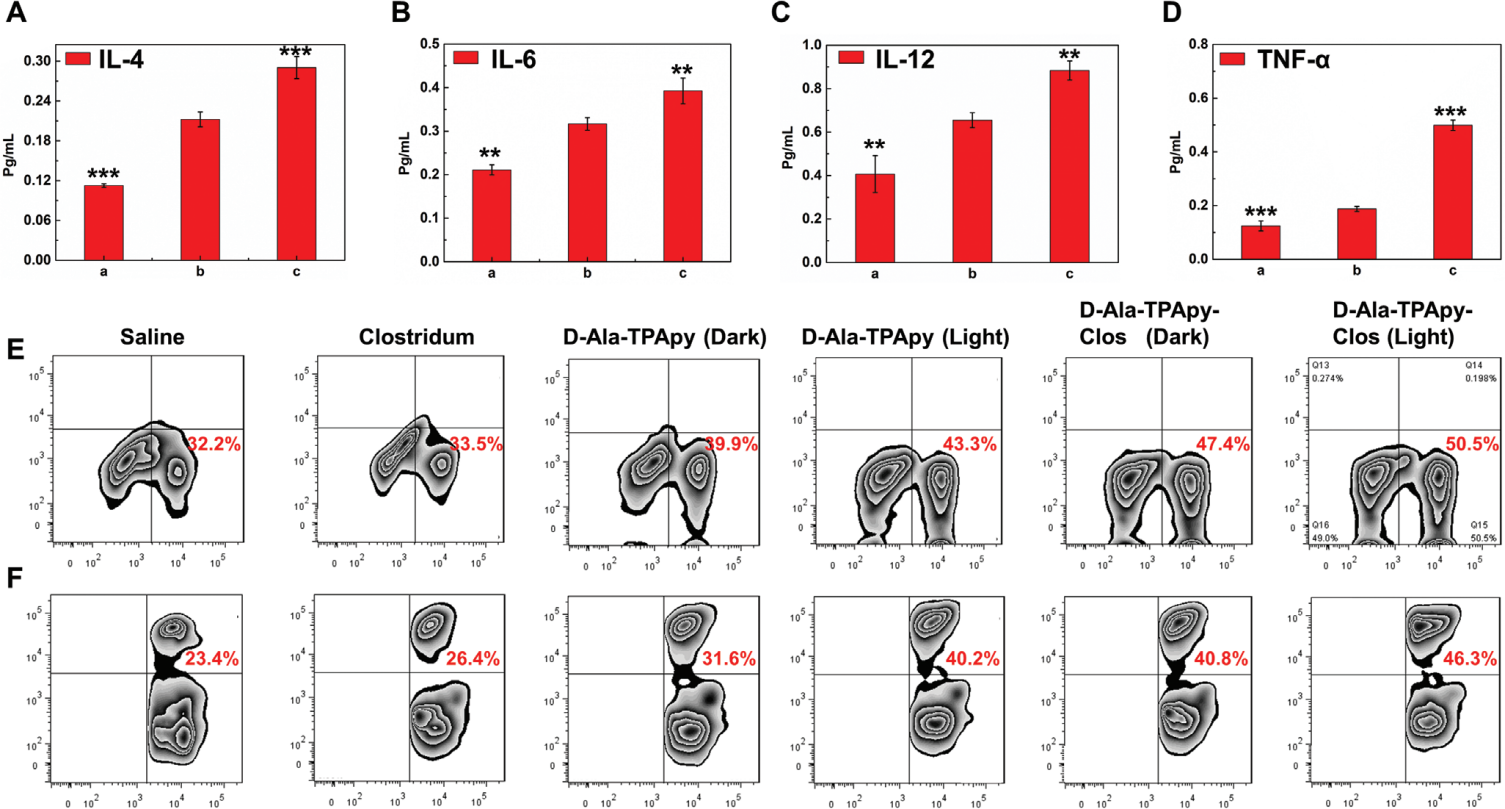
In vivo Immunology study of tumor‐bearing mice after being treated by TPApy labeled *Clostridium butyricum*. The concentration analysis of cytokine: a) IL‐4, b) IL‐6 , c) IL‐12, and d) TNF‐*α*, respectively. a) Saline group; b) d‐Ala‐TPApy‐Clos (Dark); c) d‐Ala‐TPApy‐Clos (Light). e,f) Flow cytometry analysis of CD^4+^ and CD^8+^ T cell levels of tumor tissues after being treated by different formulations. The data presented here were mean ±SD, *n* = 7. The statistical significance level is ^**^ *p* < 0.01, ^***^ *p* < 0.001.

As we all know that immune cells in the tumor microenvironment involve in tumor recurrence, progression, and metastasis. As a result, to learn the mechanism of efficient tumor inhibition, we evaluated the impact of T‐cell immune responses after intratumoral administration of d‐Ala‐TPApy‐Clos (with or without light irradiation) on the tumor microenvironment. **Figure** **5**a–d showed remarkable secretion of IL‐4, 6, 12, and interferon‐*γ* after tumor‐bearing mice were treated by d‐Ala‐TPApy‐Clos under light irradiation, indicating that CD8^+^ T‐cells were activated. We then measured treatment‐induced intratumoral infiltration of the T lymphocytes. The frequencies of the CD8^+^ T‐cells and CD4^+^ T cells were measured by flow cytometry analysis. The results showed that melanoma‐bearing mice receiving *C. butyricum* treatment had remarkable CD8^+^, CD4^+^ T‐cell infiltration in the tumor, especially being administrated by d‐Ala‐TPApy‐Clos under light (100 mW cm^−2^, 5 min) with the frequencies of the CD8^+^ T cells reaching 50.5 ± 3%. This oncolytic bacteria‐assisted immunotherapy also significantly increased the intratumoral effector CD4^+^ T‐cell ratios, demonstrating ameliorative immunotherapeutic activity within the tumor microenvironment of residual tumor. Overall, these results revealed that the oncolytic bacteria‐based treatment combination with PDT could stimulate a systematic antitumor immune response for inhibiting residual tumors and build a long‐term antitumor immune memory.

## 3 Conclusions

In summary, we developed a strategy of photosensitizer labeled anaerobic oncolytic bacteria based on metabolic engineering to achieve melanoma ablation. In contrast to traditional chemotherapy and surgical treatment, our engineered oncolytic bacteria could realize minimal invasive ablation of melanoma without drug resistance or serious side effects brought by chemotherapeutic agents. Based on the remarkable in vitro and in vivo results, PS modified oncolytic bacteria showed great promise in using live bacteria‐based biomaterials to achieve efficient and minimally invasive treatment of malignant melanoma. Further study to show the generality of the engineered functional oncolytic bacteria is ongoing, aiming to provide a useful platform for the ablation of solid tumors in the future.

## Conflict of Interest

The authors declare no conflict of interest.

## Supporting information

Supporting Information

## Data Availability

The data that support the findings of this study are available in the supplementary material of this article.
