# Supplementary material for: Living Bacteria‐Based Immuno‐Photodynamic Therapy: Metabolic Labeling of Clostridium butyricum for Eradicating Malignant Melanoma
Source: Adv Sci (Weinh). 2022 Mar 11;9(14):2105807. doi: 10.1002/advs.202105807 (PMC9108598; doi:10.1002/advs.202105807)
Supplement: Supplementary file 1 — Supporting Information [file ADVS-9-2105807-s001.pdf]

ADVANCED SCIENCE
Open Access

Supporting Information

for *Adv. Sci.*, DOI 10.1002/advs.202105807

Living Bacteria-Based Immuno-Photodynamic Therapy: Metabolic Labeling of *Clostridium butyricum* for Eradicating Malignant Melanoma

Leilei Shi, Xiaoxiao Liu, Yuzhen Li, Sha Li, Wenbo Wu, Xihui Gao and Bin Liu**

Supporting Information

Living Bacteria-Based Immuno-Photodynamic Therapy: Metabolic Labeling of Clostridium Butyricum for Eradicating Malignant Melanoma

Leilei Shi,[1,2,#] Xiaoxiao Liu,[3,#] Yuzhen Li,[2] Sha Li,[3] Wenbo Wu,[1] Xihui Gao,[3,*] and Bin Liu[1*]

[1]Dr. L. Shi, Prof. W. Wu, Prof. B. Liu, Department of Chemical and Biomolecular Engineering, National University of Singapore, 4 Engineering Drive 4, 117585, Singapore (Singapore), E-mail: cheliub@nus.edu.sg

[2]Dr. L. Shi, Y. Li, The Eighth Affiliated Hospital, Sun Yat-sen University, 3025 Shennan Middle Road, Shenzhen, China.

[3]X. Liu, S. Li and Prof. X. Gao, Key Laboratory of Medical Molecular Virology (MOE/NHC/CAMS), School of Basic Medical Sciences, Fudan University, 131 Dong An Road, 200032, Shanghai, China, E-mail: gaoxihui@fudan.edu.cn

Supplementary Experimental Section:

General methods, materials, and instruments

All reactions were carried out under Argon atmosphere if no additional indication. All solvents were processed according to standard methods before use and reagents were used as purchased without purification. 5-formyl-2-thienylboronic acid, [1,1′-bis(diphenylphosphino)ferrocene] dichloropalladium (II), piperidine, propargyl bromide solution, D-alanine, copper (I) bromide, K_2CO_3, 4-methylpyridine, dichloromethane (DCM), anhydrous dimethyl sulfoxide (DMSO), toluene, methanol, ethanol, chloroform, 3-(4,5-dimethylthiazol-2-yl)-2,5-diphenyltetrazolium bromide (MTT), anhydrous *N, N*-dimethylformamide (DMF) and all other materials for organic synthesis were purchased from Sigma-Aldrich and used without further purification. Dulbecco’s Modified Eagle Medium (DMEM) was purchased from Gibco. Fetal bovine serum (FBS), penicillin, and streptomycin were obtained from Gibco. TNF-α, IL-12, and IFN-γ ELISA kits were purchased from Life technologies, USA. The bacteria (clostridium butyricum) were purchased from ATCC.

^{1}H-NMR spectra were recorded on 400 MHZ Bruker® and ^{13}C-NMR spectra were recorded on 100 MHz Bruker®. Data for ^{1}H-NMR are recorded as follows: chemical shift (δ, ppm), multiplicity (s = singlet, d = doublet, t = triplet, q = quartet, m = multiplet or unresolved, coupling constant(s) in Hz, integration). Data for ^{13}C-NMR are reported in terms of chemical shift (δ, ppm). The measurement of UV-vis absorption spectra was carried out using a UV-vis absorption spectrometer (Shimadzu, UV-1700, Japan). PL spectra were collected using a Perkin-Elmer LS-55 equipped

with a xenon lamp excitation source and a Hamamatsu (Japan) 928 PMT, using 90° angle detection for solution samples. The *in vitro* cell imaging and immunofluorescence were measured by laser confocal fluorescence microscopy (TCS SP8 STED 3X, Leica, Instrumental Analysis Center of Shanghai Jiao Tong University).

Synthesis of 1-(but-3-yn-1-yl)-4-methylpyridin-1-ium (compound 1)

(4-Methylpyridine (465 mg, 5.0 mmol) was dissolved in 15 mL MeCN, propargyl bromide solution (1.98 g, 15.0 mmol) was added and the reaction mixture was heated to reflux overnight. After completion, the solvent was removed and the crude product was precipitated from EtO_2. The solid was filtered to give compound 1 (646.6 mg, 61% yield) as a brown solid. ^{1}H NMR (400 MHz, DMSO) δ 8.99 - 8.97 (d, 2H), 8.05 - 8.03 (d, 2H), 5.57 (d, 2H), 4.05 (m, 1H), 2.64 (s, 3H).

Synthesis of (E)-1-(but-3-yn-1-yl)-4-(2-(5-(4-(diphenylamino)phenyl)thiophen-2-yl)vinyl)pyridin-1-ium (compound 2)

A solution of compound 1 (54.48 mg, 0.3 mmol) and 5-(4-(diphenylamino)phenyl)thiophene-2-carbaldehyde (103.4 mg, 0.4 mmol) was refluxed under nitrogen in dry ethanol catalyzed by a few drops of piperidine overnight. After cooling to room temperature, the mixture was poured into diethyl ether. The residue was dissolved in DCM and washed by water. The combined organic phase was dried over anhydrous sodium sulfate and concentrated under reduced pressure to give a residue which was purified by preparative thin layer

chromatography (methanol/dichloromethane = 1/20, V/V) to give the desired product compound 4 (66.1 mg, 39 % yield) as red solid. ^{1}H NMR (400 MHz, DMSO) δ 8.73 (d, J = 8.0 Hz, 2H), 8.14 - 8.08 (m, 3H), 7.59 (d, J = 8.0 Hz, 2H), 7.48-7.46 (m, 1H), 7.39-7.38 (m, 1H), 7.33-7.29 (m, 4H), 7.11-7.01 (m, 7H), 7.04 - 7.01 (m, 2H), 4.63 (t, J = 8.0 Hz, 2H), 2.94 (t, J = 8.0 Hz, 2H), 2.57 (s, 1H). ^{13}C NMR (100 MHz, $CDCl_3$, 298K) δ (ppm): 19.9, 57.8, 72.5, 95.4, 120.2, 122.4, 122.9, 123.5, 124.9, 126.4, 126.8, 129.1, 134.2, 134.9, 138.7, 143.5, 147.1, 148.7, 149.2, 154.7.

Synthesis of Final product D-Ala-TPApy (compound 3)

Compound 2 (56.5 mg, 0.1 mmol) and 3-azido-D-alaninehydrochloride (13 mg, 0.10 mmol) were dissolved in argon degassing DMSO (2.0 mL) in a 5 mL round bottom flask. Copper (I) bromide (9.4 mg, 0.066 mmol) was dispersed in 0.2 mL trimethylamine and injected into the solution. The reaction was stirred at room temperature overnight. Then the mixture was filtrated by polyethersulfone 0.45 μm membrane filters and subjected to HPLC for purification. HPLC conditions: column: Agilent ZORBAX SB-C18 (9.4×150 mm); gradient: 0-6-13-17 min, 25%-80%-100%-100% B (A: H2O containing 0.1% TFA, B: acetonitrile containing 0.1% TFA); flow rate is 2.0 mL min-1; UV-Vis detector: 254 nm. Compound 3 was obtained as red powder (18.3 mg, 30% yield). ^{1}H NMR (400MHz, DMSO) δ ^{1}H NMR (400 MHz, DMSO) δ 8.81 (d, J = 8.0 Hz, 2H), 8.51 (s, 2H), 8.23-8.19 (m, 2H), 8.14 (d, J = 8.0 Hz, 2H), 7.94 (s, 1H), 7.65-7.62 (m, 2H), 7.53-7.50 (m, 2H), 7.39-7.35 (m, 4 H), 7.15-7.09 (m, 6H), 6.99 (d, J = 8.0 Hz, 2H), 4.83-4.82 (m, 2H), 4.78-4.77 (m, 2H), 4.55 (s, 1H), 3.88 (m, 2H), 3.37 (t, J = 8.0 Hz, 2H). ^{13}C NMR (100 MHz, $CDCl_3$,

298K) δ (ppm): 26.6, 48.6, 52.0, 58.9, 121.3, 121.7, 122.8, 123.4, 124.4, 124.7, 124.8, 125.2, 126.4, 126.5, 127.5, 130.5, 134.2, 134.6, 138.7, 142.4, 144.6, 146.7, 147.7, 148.4, 152.9, 168.8. HR-ESI-MS, m/z: $[M]^+$ calcd 678.1896, found 678.1406.

Overall ROS Detection in Aqueous Solution

A commonly used ROS indicator DCFH-DA was utilized to detect the ROS generation of TPApy aqueous solution under light irradiation (100 mW/cm^2). DCFH-DA was prehydrolyzed into DCFH according to the protocol. Then, the ROS indicator (40×10^{-6} M) in PBS was further diluted to 5×10^{-6} M in the sample solution of TPApy for measurement by PL instrument. The fluorescence of 2′,7′-dichlorofluorescein in triggered by TPApy-sensitized ROS under white light irradiation was measured at different time intervals. The PL spectra were measured with excitation at 488 nm and emission was collected from 500 to 620 nm. The fluorescence intensity at 525 nm was recorded to indicate the generation rate of overall ROS.

•OH Detection in Aqueous Solution

The •OH generation potency was evaluated by using HPF as an indicator. The stock solution of HPF (5×10^{-3} M in *N*, *N*-dimethylformamide) was diluted to 5×10^{-6} M in the sample solution of TPApy in PBS buffer. The fluorescence signal of HPF was monitored at different time intervals in a range of 500–620 nm with the excitation wavelength at 490 nm after the solution was irradiated by light irradiation (100 mW/cm^2). The fluorescence intensity at 515 nm was recorded to indicate the generation rate of •OH.

•O^{2-} Detection in Aqueous Solution

The •O^{2-} generation measurements were performed using DHR123 and HKSOX-1 as indicators. The stock solution of DHR123 and HKSOX-1 were diluted to working concentration according to the protocol. And then, being mixed with TPApy in PBS buffer. The fluorescence signal of DHR123 and HKSOX-1 was monitored at different time intervals after the solution was irradiated by light irradiation (100 mW/cm^2).

Preparation of the D-Ala-TPApy-labeled Clostridium butyricum

The Clostridium butyricum cells were routinely grown at 37 °C and 220 rpm in LB medium. After overnight culture, the bacteria were collected and the optical density at 600 nm (OD600) was adjusted to 1.0 in PBS. The bacterial suspension was added into D-Ala-TPApy (0 ~ 50) μM solutions with the same volume, mixed and co-cultured at 37 °C for 30 min. The mixtures were centrifuged at 6000 rpm for 4 min and the supernatants were discarded, washed twice and the D-Ala-TPApy-labeled bacteria were obtained.

Characterization of peptidoglycan fragment from Clostridium butyricum

Overnight pre-cultured bacterial cells were added into a new LB medium and the bacteria were incubated on a shaker until reaching the log phase. Then, 5 mL of the bacteria were obtained by centrifugation and re-suspended in 5 mL of LB medium containing 50 μM D-Ala-TPApy and the mixture was incubated for 2 h. After that, the labeled bacteria were obtained by centrifugation at 5,000 g for 10 min and they were quickly cooled on the ice for 15 min and subsequently re-suspended in 4 mL water. The cells were washed once with 5 mL of PBS. The cell pellets were re-suspended in

5 mL of PBS for protecting the acetyl groups in bacteria. Then, the bacterial suspension was added into sodium dodecyl sulphate (SDS, 8% w/v) with the same volume and the mixture was boiled for 4 h under stirring. The samples were equilibrated to room temperature and collected with a tabletop centrifuge at 30,000 g for 15 min at room temperature and washed with water three times to remove SDS and pellet the insoluble peptidoglycan. The pellet peptidoglycan was dispersed in a Tris buffer (pH = 7.5) with a concentration of 5 mM Tris-HCl, 10 mM NaCl and 100 μg/mL α-amylase and incubated for 2 h at 37 °C. The samples were then collected and dispersed in 10 mM Tris-pH 7.5 with 200 μg/mL pronase and incubated for 2 h at 37 °C under agitation. The samples were obtained and dispersed in 1 mL of water and added into the same volume of 4% SDS and boiled for 30 min with stirring. The samples were equilibrated to room temperature and collected with a tabletop centrifuge at 30,000 g for 15 min at room temperature and were washed with water three times to remove SDS. The purified peptidoglycan was transferred to 10 mM Tris-pH 7.5 with 2 mg/mL of mutanolysin and incubated at 37 °C for 2 h under agitation. Centrifugation at 15,000 g for 15 min was performed to remove the mutanolysin and insoluble peptidoglycan. The supernatant containing soluble peptidoglycan was collected for matrix-assisted laser desorption ionization-time of flight mass spectrometry (MALDI-TOF MS) analysis.

In vitro ROS detection by ABDA:

The ROS was detected using ABDA as the ROS indicator. 5 μL of ABDA stock solution was added into 1 mL of sample solution (10 μM), and the mixtures were

irradiated by white light (400 - 800 nm) at a power density of 30 mW cm^{-2}. The absorbance of ABDA was monitored at different exposure times at 378 nm to obtain the decay rate of the photosensitizing process.

Cell culture

The melanoma cell line B16F10 was cultured in Dulbecco's Modified Eagle Medium (DMEM). The culture medium contains 10% fetal bovine serum (FBS) and antibiotics (50 units mL^{-1} and 50 units mL^{-1} streptomycin) at 37°C under a humidified atmosphere containing 5% CO_2 for 24 h and then replaced the fresh medium.

Cell uptake study of D-Ala-TPApy-labeled Clostridium butyricum

The cellular uptake of D-Ala-TPApy-labeled Clostridium butyricum in B16F10 cell lines was evaluated. B16F10 were plated on 8-well plates at a density of 5×10^4/well and grown in DMEM supplemented with 10% fetal bovine serum (FBS) (Thermo Fisher Scientific Inc., USA) and 10 μg/mL gentamicin (Thermo Fisher Scientific Inc, USA) for 20 h at 37 °C at a humidified 5% CO_2 incubator. The medium was replaced with 300 μL of fresh medium and 100 μL of D-Ala-TPApy-labeled Clostridium butyricum at bacteria/cell ratio of 20 were added to each well. After 2 h incubation, the medium was discarded. After 2 hours, the medium was replaced by media containing the penicillin streptomycin solution for another 2 h incubation to eliminate extracellular bacteria. The cells were then washed twice with PBS and then treated with 10 μg/mL of Hoechst 33342 in PBS for 10 min. The images were taken on a confocal scanning laser microscope. The fluorescence of Hoechst was excited with a 405 nm laser with emission collected at 420-450 nm. The fluorescence of

D-Ala-TPApy was excited with a 488 nm laser with emission collected at 650-750 nm and observed by (Leica)/TCS SP8 STED 3X.

***In vitro* cytotoxicity study**

For cytotoxicity study, B16F10 cells were seeded in 96-well plates. Twelve hours later, cells were treated with different groups at various concentrations with or without light irradiation (100 mW/cm^2, 5 min) and incubated for 72 h. Then, 20 μL of MTT solutions (5 mg/mL) were added to the 96-well plates. After 4 h incubation at 37°C, the medium was replaced with 200 μL of DMSO. The obtained solution was measured in a Bio Tech Synergy H4 at a wavelength of 570 nm.

Animals and tumor models

Experiment protocols involving animals were authorized by the Animal Ethics Committee of the School of Medicine, Fudan University. C57 BL/6 male mice (4 weeks of age) were purchased from the Chinese Academy of Science (Shanghai). The male mice were injected subcutaneously with 100 μL of cell suspension containing 5×10^5 B16F10 cells. The tumor volume was allowed to grow to about 100 mm^3 before *in vivo* experiment.

***In vivo* pharmacodynamic study**

The melanoma-bearing C57 BL/6 mice were randomly divided into six groups (n = 8). Mice in various groups were one-time intra-tumoral injection different agents with blue light irradiation (100 mW/cm^2) for 5 min or without light illumination for 9 days. The length and width of the tumor and the bodyweight of mice were measured before every injection during the therapy. The tumor volume was calculated using the

formula

$$V\ (mm^3) = 1/2 \times \text{length (mm)} \times \text{width (mm)}^2$$

After 9 days' treatment, some of the mice were sacrificed and tumors were separated, weighed, and photographed. Additionally, the tumors were cut into small pieces, fixed in 40% formalin, and embedded in the paraffin. Then, the tissues were sectioned for histopathological analysis with H&E staining, immunohistochemical analysis, and TUNEL immunofluorescence assay. For histology analysis, tissues were fixed in 4% paraformaldehyde and embedded in paraffin. Paraffin-embedded serial sections (5 μm) were obtained, stained with hematoxylin solution and eosin Y solution (H&E) to assess histological alterations by microscope (Olympus). In immunohistochemical studies, the paraffin embedded tumor sections were mounted on glass slides, heated for 30 min at 60 °C. Followed by incubations in a bath at room temperature, the deparaffinized slides were rehydrated by graded alcohols (100%, 95%, 85%, 75%) and washed in distilled water. Pretreated with blocking medium for 5 min, the slides were incubated with primary anti-HIF 1α antibody (1:500, ABcam) in antibody diluent solution for 30 min at room temperature and then 4 °C for 12 h. After washing with distilled water (1 mL) three-time, the slides were counterstained using Aquatex (Merck, Germany) and observed by microscope (Olympus). Tissues apoptotic cells were assessed by a terminal transferase dUTP nick-end labeling (TUNEL) assay kit (In Situ Cell Death Detection Kit, Roche, USA) according to the manufacturer's protocol. The paraffin-embedded 5-μm-thick tumor sections were mounted on glass slides, heated for 30 min at 60 °C. Followed by incubations in a bath at room

temperature, the deparaffinized slides were rehydrated by graded alcohols (100%, 95%, 85%, 75%) and washed in distilled water. The slides were treated with freshly prepared 2% H_2O_2 for 10 min at room temperature and washed three times with distilled H_2O for 2 min. Then slides were covered by 20 μg/mL of proteinase K solution (1 mL) for 15 min at room temperature and washed with distilled H_2O three times subsequently. The slides were blotted away excess water carefully, and then incubated with TdT equilibration buffer (2.5 mM Tris-HCl (pH 6.6), 0.2 M potassium cacodylate, 2.5 mM $CoCl_2$, 0.25 mg/mL bovine serum albumin (BSA)) for 10 min at room temperature. After removing the TdT equilibration buffer, the slides were incubated with TdT reaction buffer for 30 min at 37°C. TdT buffer was carefully covered with a glass coverslip and incubated for 30 min at room temperature. The slides were incubated in 2×SSC (300 mM NaCl, 30 mM sodium citrate) for 10 min to stop the reaction. After washing with 1× PBS (1 mL), the slides were incubated with 2% BSA solution for 30-60 min at room temperature. After washing with 1× PBS (1 mL) again, the slides were then covered with TdT staining buffer for 30 min in the dark. After being washed with PBS and stained with Hoechst 33342, the slides were washed with PBS again, dried in the air, and attached coverslips by Vectashield antifade mounting medium. The tissue sections were examined by laser confocal fluorescence microscopy (Leica) at an excitation wavelength of 488 nm with an emission wavelength of 510-550 nm. Hoechst 33342 was excited with a 405 nm laser with emission collected at 420-460 nm.

The analysis of T cells and cytokine secretion in vivo

The male C57 BL/6 mice harboring B16F10 tumor model were used to evaluate for the influence of $CD4^+$, $CD8^+$ T cells, and cytokines. Briefly, after various treatments, five mice per group were sacrificed and the tumors, spleens, and blood were harvested for detection. The tissues were digested with collagenase IV (175 U/mL), hyaluronidase (100 U/mL), and DNase (30 U/mL) at 37 °C for 60 min. The tumor-infiltrating lymphocytes (TILs) were isolated by passing 75 μm filters and enriched for the following analysis. For the T cells frequency detection, the TTLs were labeled with anti-CD3-APC, anti-CD4-FITC, and anti-CD8-PE. The stained TILs were then washed thrice with PBS and analyzed via flow cytometry.

For cytokine quantification, the harvested spleen cells were suspended in 5 mL of the DMEM culture medium supplemented with 10 % FBS for 24 h. The supernatants were collected and cytokines were quantified using mouse IL-4, IL-6, IL-12, and IFN-γ ELISA kits (Life technologies, USA).

Statistics study

Quantitative data were presented as mean ± standard error of the mean (SEM). ANOVA analysis was used for multiple comparisons, and Student's t-test was used for two-group comparisons. Sample size (n) for in vitro and in vivo studies was 3 and 7, respectively. The differences in each group were analyzed by the Kaplan–Meier method, and the P-value was determined by the log-rank test. All statistical analyses were carried out using GraphPad Prism 5.0. (P-value: $*P < 0.05$, $**P < 0.01$, $***P < 0.001$).

References

[S1] W. Wu, D. Mao, X. Cai, Y. Duan, F. Hu, D. Kong, B. Liu, *Chem. Mater.* **2018**, *30*, 3867-3873.

Supplementary Figures:

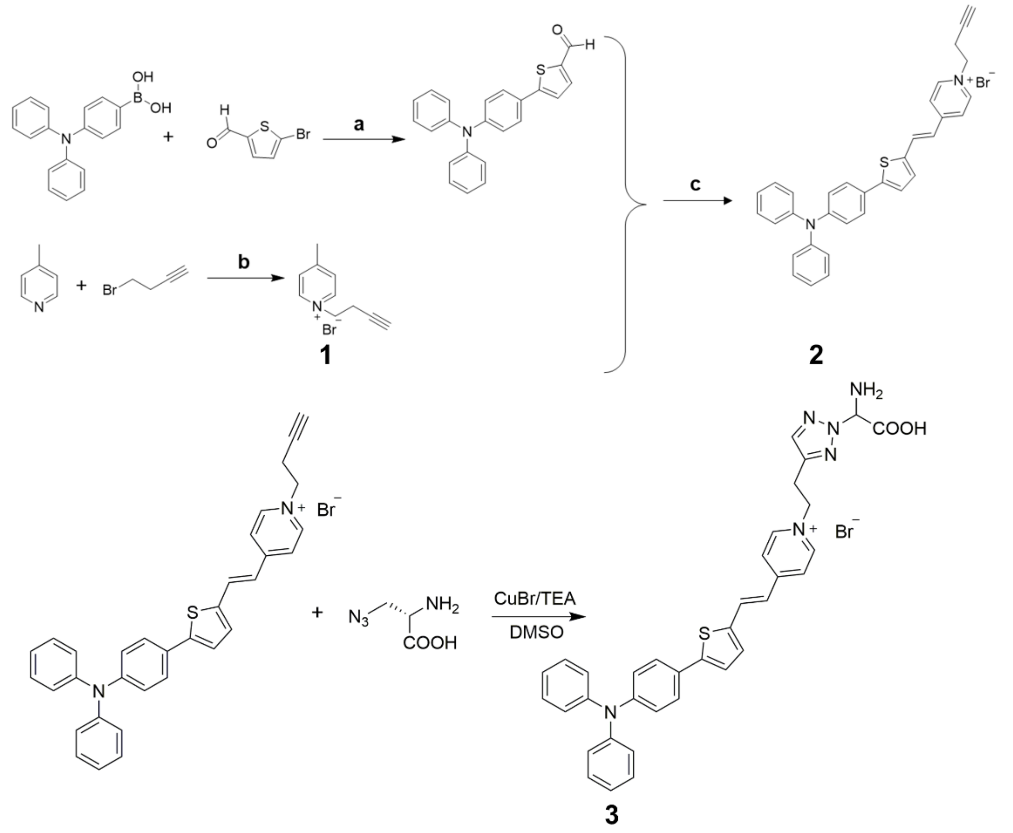

Scheme S1: The synthetic route of the metabolic substrate D-Ala-TPApy (compound 3). a, Pd(dppf)Cl_2, K_2CO_3, MeOH/ toluene, reflux; b, CH_3CN, reflux; c, Ethanol,

piperidine, N_2; d, CuBr, TEA, DMSO, Ar.

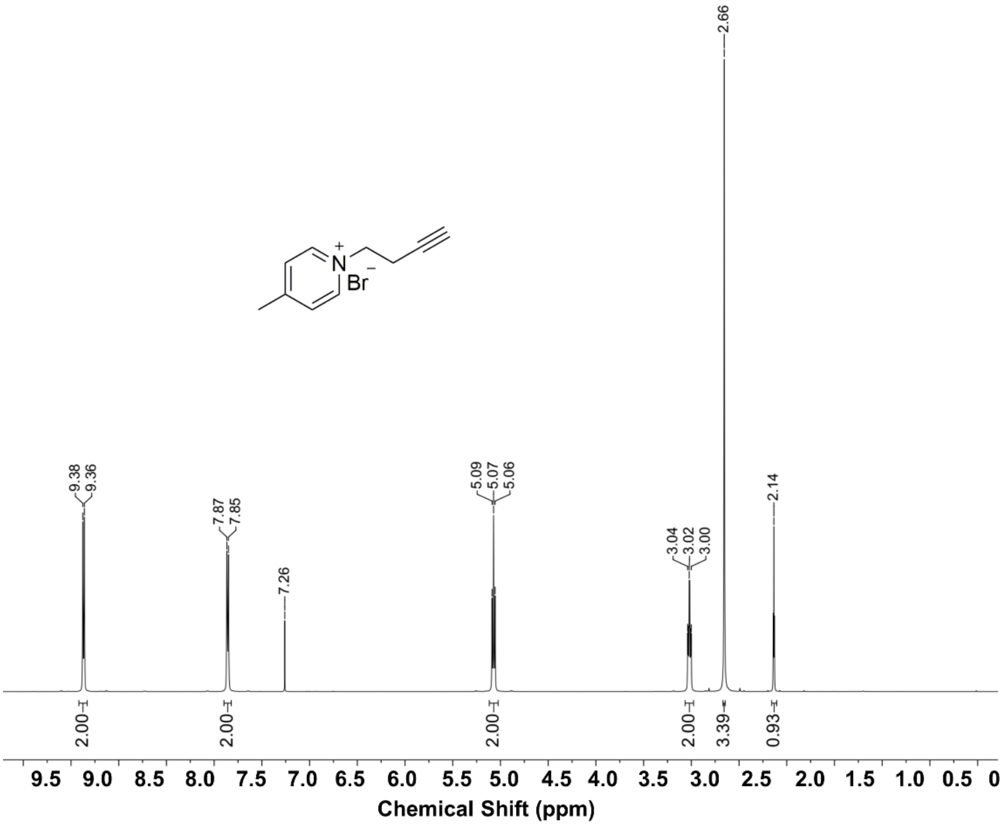

Figure S1. ^{1}H NMR spectrum of compound 1 ($CDCl_3$).

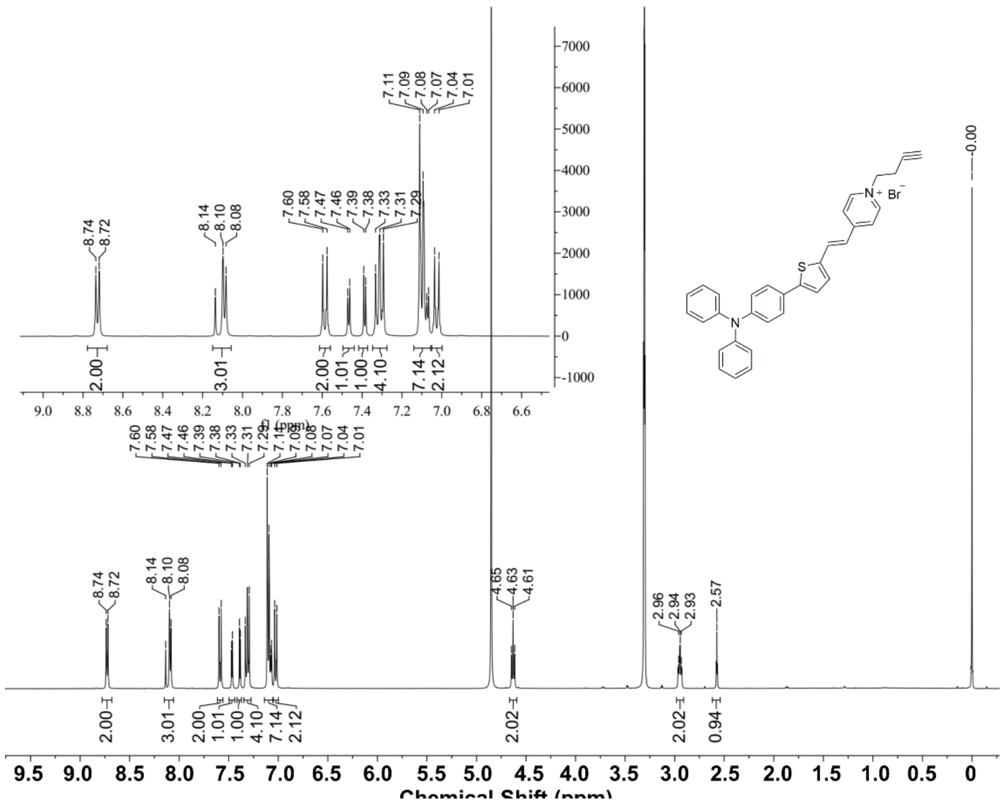

Figure S2. ^{1}H NMR spectrum of compound 2 (CD_3OD).

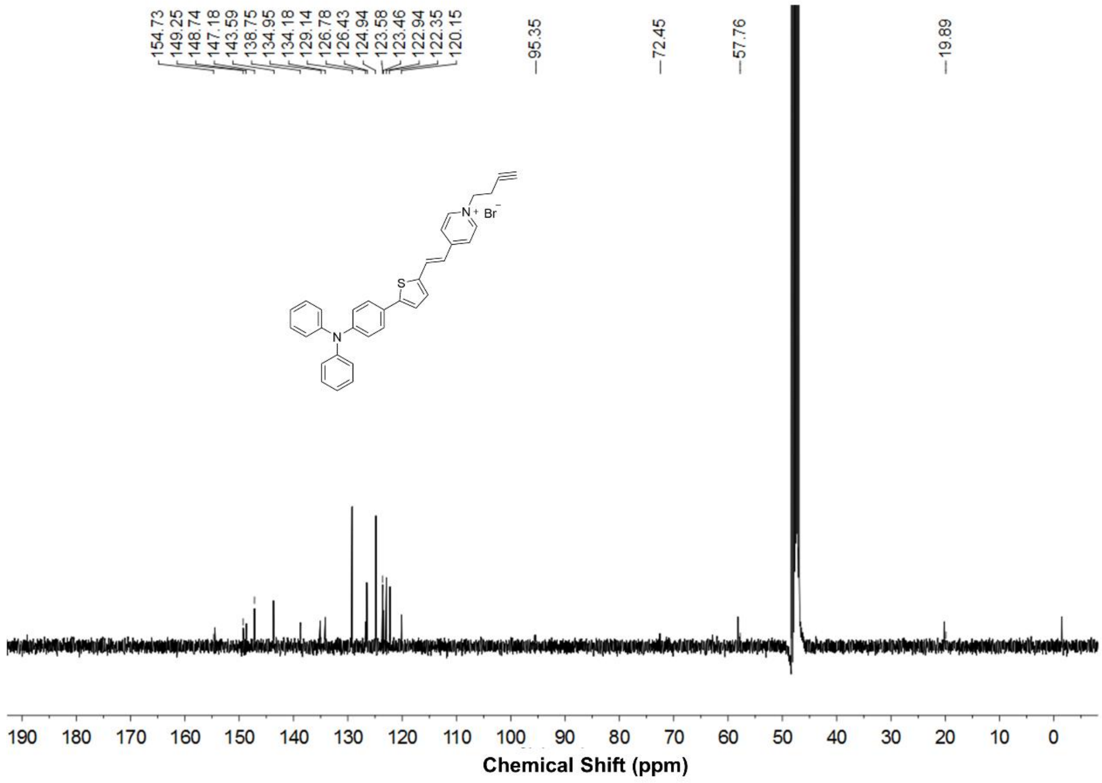

Figure S3. ^{13}C NMR spectrum of compound 2 (CD_3OD).

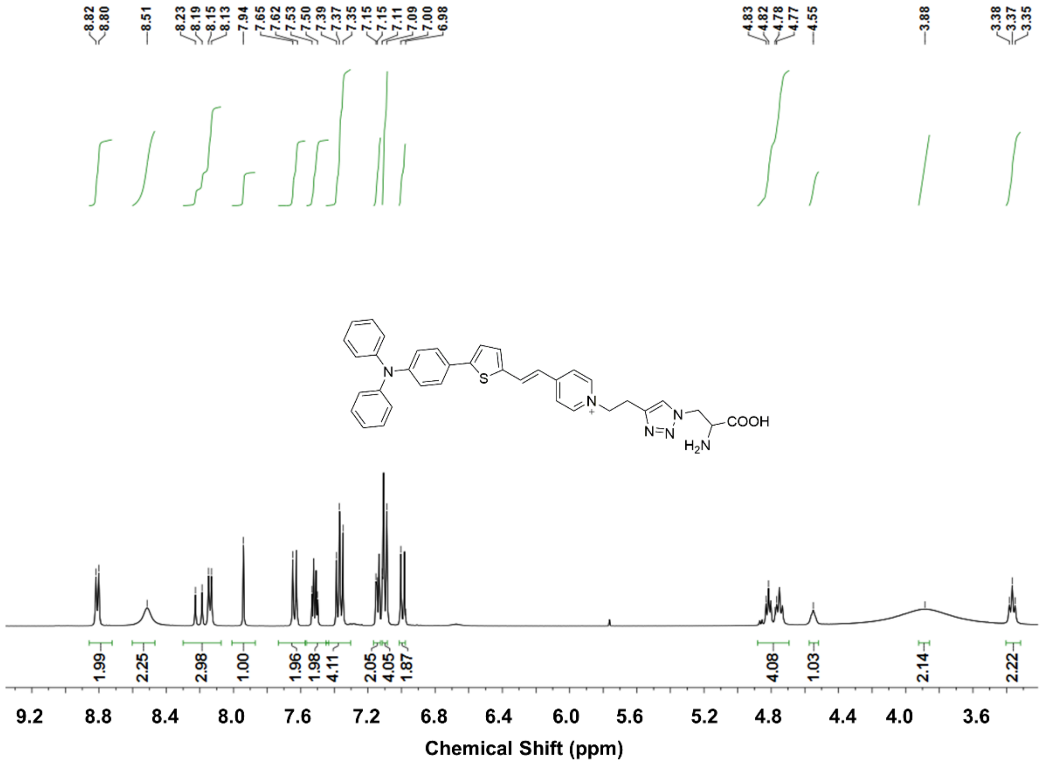

Figure S4. ^{1}H NMR spectrum of final product D-Ala-TPApy (compound 3) (DMSO-d^{6}).

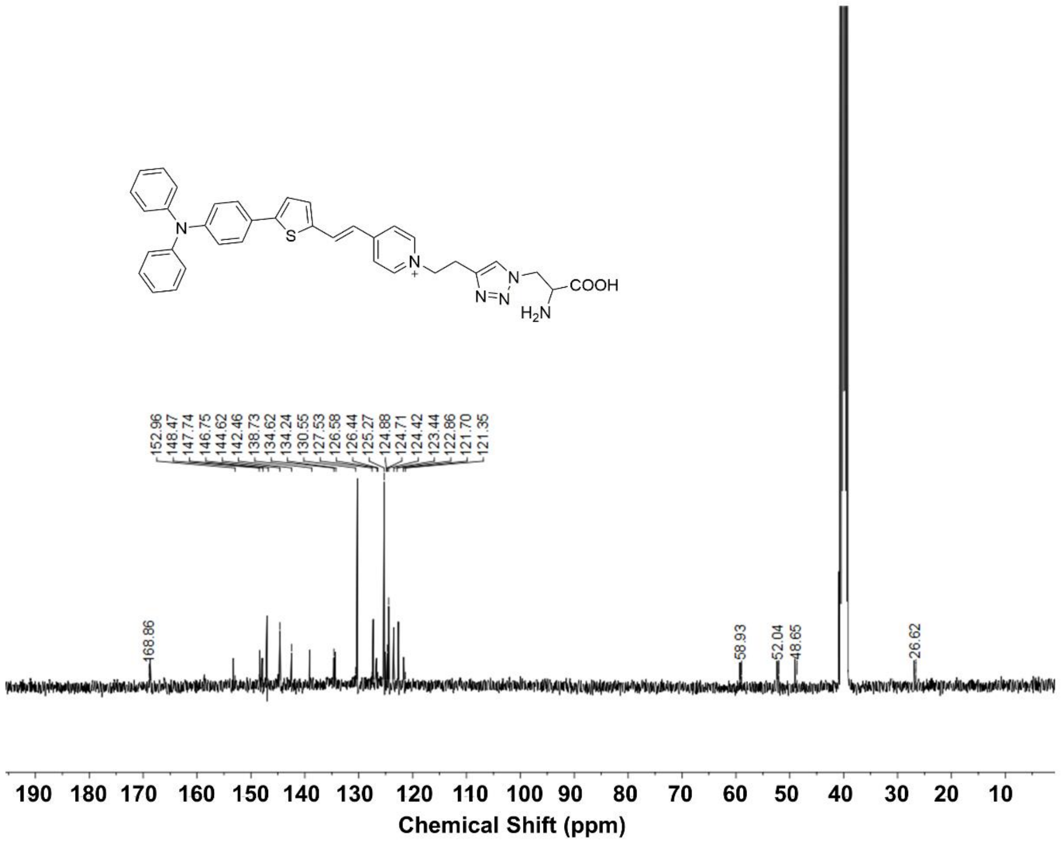

Figure S5. ^{13}C NMR spectrum of final product D-Ala-TPApy (compound 3) (DMSO-d^6).

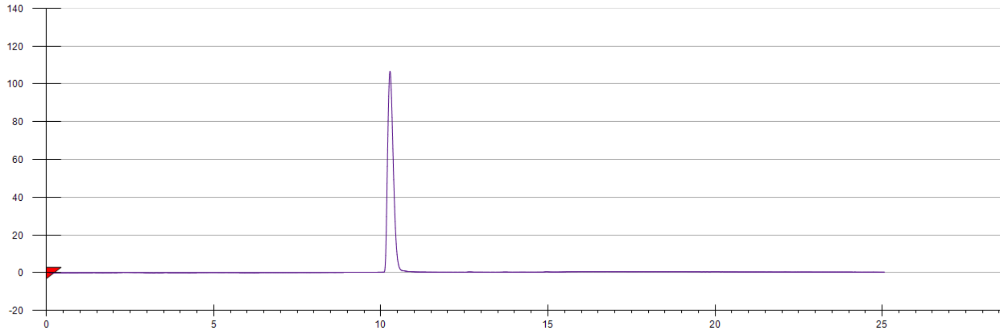

Figure S6. The purity analysis of D-Ala-TPApy through high performance liquid chrmatography.

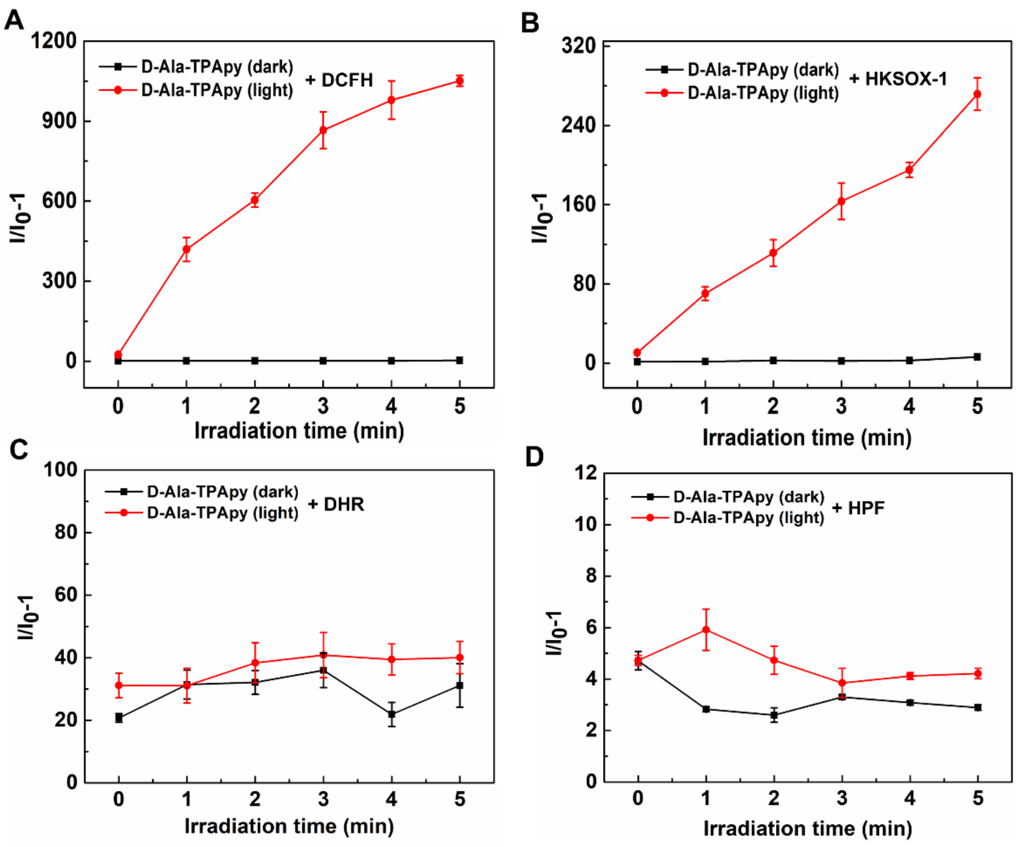

Figure S7. ROS generation of D-Ala-TPApy upon light irradiation. Relative changes in PL intensity of A) DCFH (for overall ROS detection); B) HKSOX-1 (for O2•− detection) in the presence of D-Ala-TPApy upon light irradiation for different times; C) Relative changes in PL intensity of DHR (for ROS detection); D) HPF (for •OH detection) in the presence of D-Ala-TPApy upon light irradiation for different times.

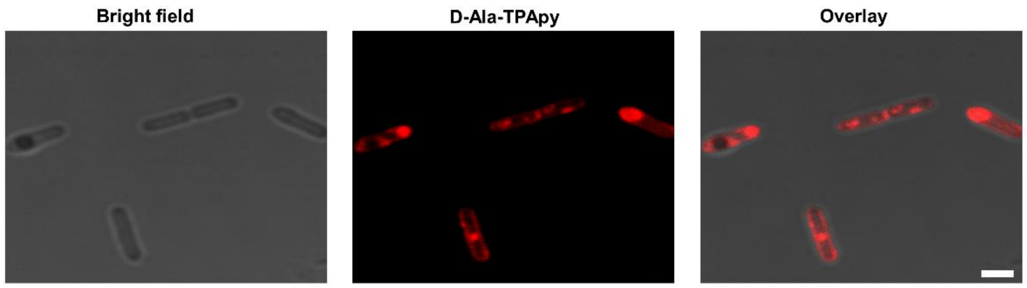

Figure S8. Confocal fluorescence images of Clostrsidum butyricum after incubation with D-Ala-TPApy (TPApy = 20 µM, 500 µL) for 0.5 h. The red fluorescence is from TPApy (Ex: 488 nm; Em: 680-750 nm). The scale bar is 0.5 µm.

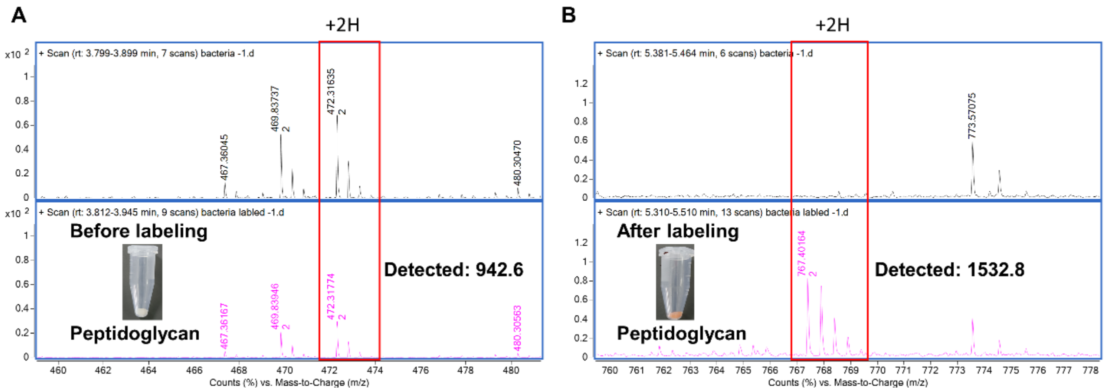

Figure S9. MALDI-TOF MS of Clostridium butyricum and D-Ala-TPApy treated Clostridium butyricum. A. Clostridium butyricum without metabolic labeling; B. Clostridium butyricum with D-Ala-TPApy metabolic labeling.

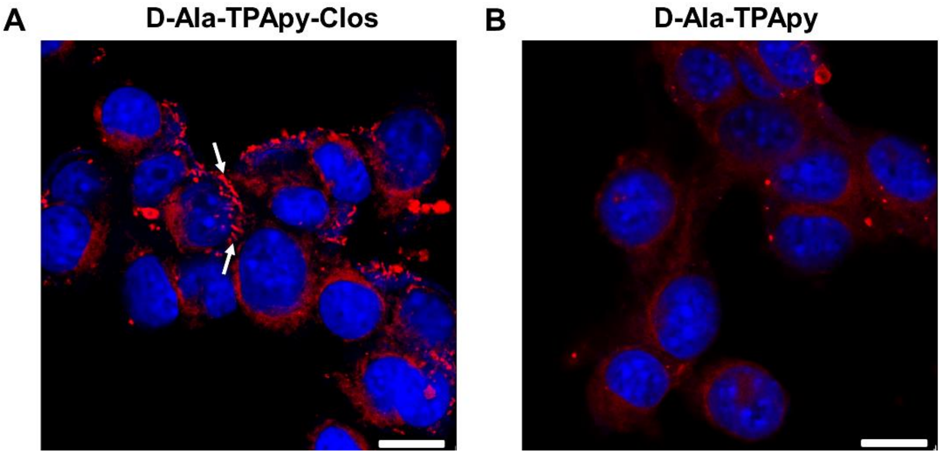

Figure S10. Confocal fluorescence images of live B16F10 after treatment with D-Ala-TPApy labeled Clostrsidum butyricum (A) and free D-Ala-TPApy (B) for 8 h. [D-Ala-TPApy] = 10 μM, 500 μL. The scale bar in all the images is 10 μm. The white arrows indicate D-Ala-TPApy-labeled Clostrsidum butyricum.

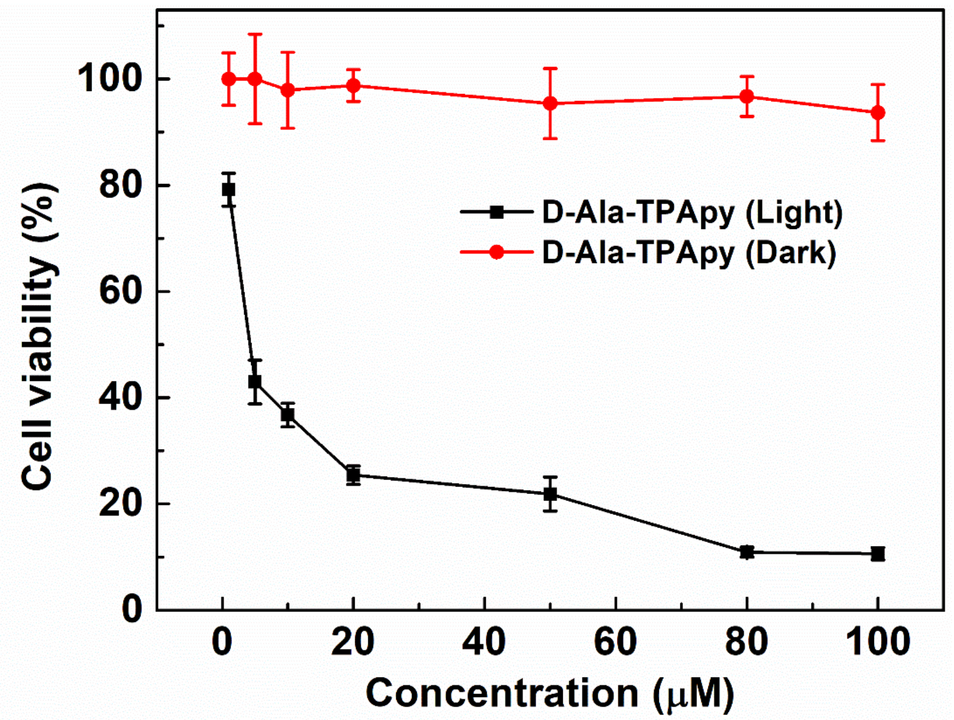

Figure S11. Cytotoxicity study of B16F10 cell lines after being treated by D-Ala-TPApy under dark and light illumination (100 mW/cm^2), respectively.

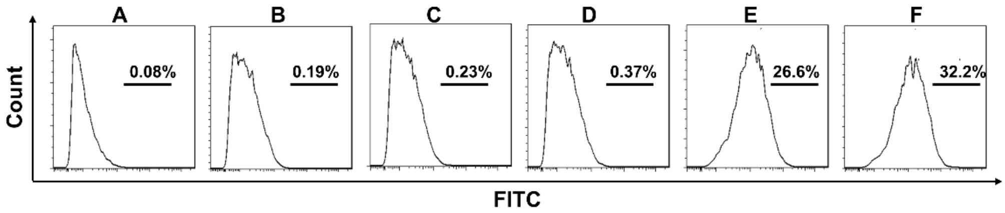

Figure S12. Flow cytometry study of intracellular ROS concentration after cells being treated with different agents for 4 h. Then, cells were further incubated with 20 μM of DCFDA for 30 min to determine intracellular ROS (FITC channel). A: saline group; B: D-Ala-TPApy (20 μM, Dark); C: Clostridium; D: TPApy (20 μM) labeled Clostridium butyricum (Dark); E: D-Ala-TPApy (20 μM, Light); F: TPApy (20 μM) labeled Clostridium butyricum (Light).

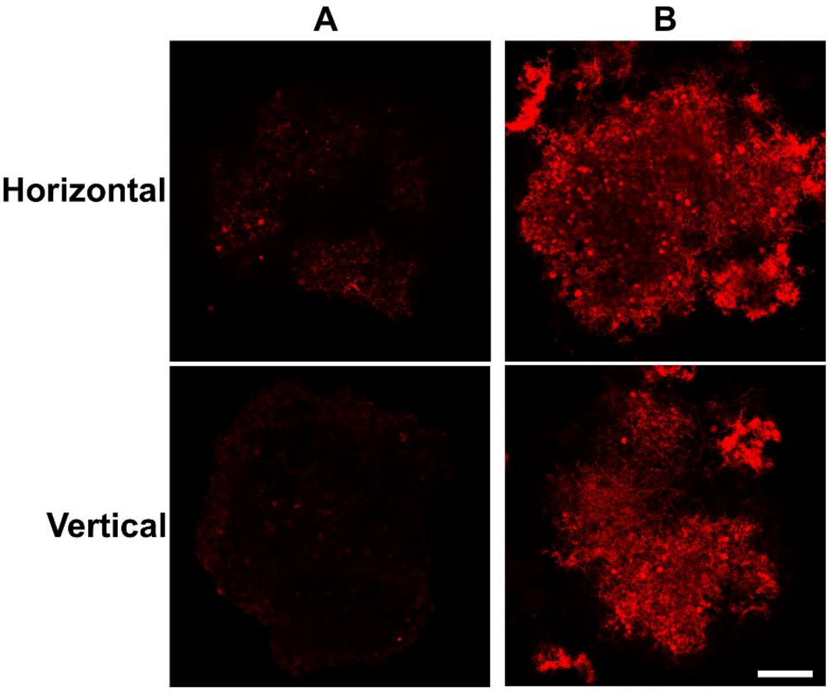

Figure S13. Confocal image study of 3D tumorspheres for tumor penetration evaluation after tumorspheres being incubated with D-Ala-TPApy (20 μM) (A) and TPApy (20 μM) labeled Clostridium butyricum (B) and viewed from horizontal and vertical cross-sections. All the images in each figure share the same scale bar, 50 μm.

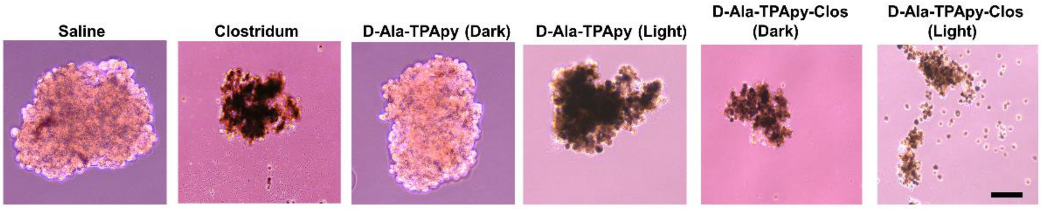

Figure S14. Size shrinkage of 3D tumorspheres treated by different agents over 7 days. All the images in each figure share the same scale bar, 50 μm. The data presented here was average tumor 3D sphere inhibition rate ± SD, n = 3.

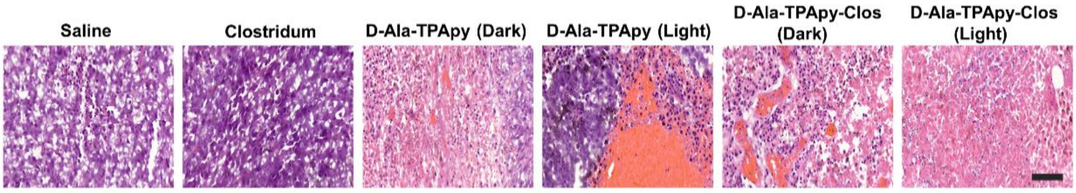

Figure S15. H&E staining assay of tumor tissues after tumor-bearing mice being treated by different agents. The scale bar is 50 µm.

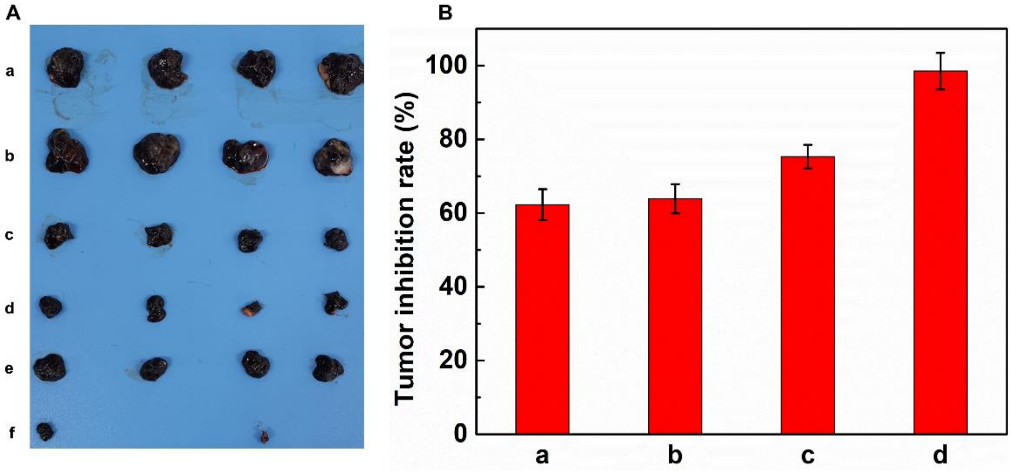

Figure S16. In vivo tumor inhibition rate study after tumor-bearing mice being treated by different formulations. A: a) saline; b) D-Ala-TPApy (Dark); c) D-Ala-TPAPy-Clos (Dark); d) D-Ala-TPAPy (Light); e) clostridium; f) D-Ala-TPAPy-Clos (Light). B: a) D-Ala-TPAPy-Clos (Dark); b) D-Ala-TPAPy (Light); c) Clostridium; d) D-Ala-TPAPy-Clos (Light). The data presented here was average tumor inhibition rate ± SD, n = 7.

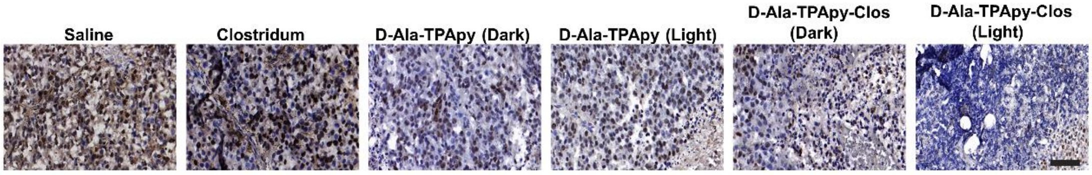

Figure S17. *In vivo* immunohistochemistry (IHC) analyses of Ki67 expression level in tumor tissue sections after tumor-bearing mice being treated by different agents. The scale bar is 50 µm.

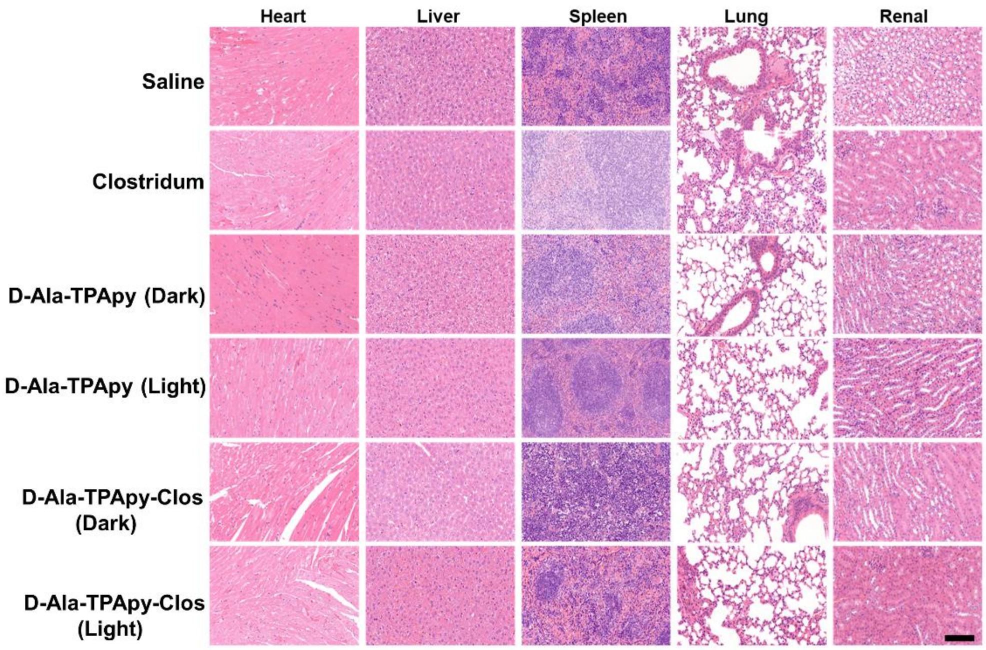

Figure S18. Microscopic images of H&E-stained sections of the major organs (heart, liver, spleen, lung, kidney) after tumor-bearing nude mice being treated with different formulations. The scale bar is 50 μm.
